# Nutritional load in post-prandial oxidative stress and the pathogeneses of diabetes mellitus

**DOI:** 10.1038/s41538-024-00282-x

**Published:** 2024-06-27

**Authors:** Fangzhou He, Junshi Liu, Yuanding Huang, Lan Chen, Ehsan Parvaresh Rizi, Ke Zhang, Lijing Ke, Tze Ping Loh, Meng Niu, Weng Kung Peng

**Affiliations:** 1 Songshan Lake Materials Laboratory, Dongguan, China; 2 Dongguan Institute of Technology, Dongguan, China; 3 BioSyM, SMART Centre, Singapore, Singapore; 4 National University of Singapore, Singapore, Singapore; 5 School of Food Science and Nutrition, University of Leeds, Leeds, UK; 6 National University of Health System, Singapore, Singapore; 7 Department of Interventional Radiology, Shengjing Hospital of China Medical University, Shenyang, China

**Keywords:** Diabetes complications, Nutrition, Pre-diabetes

## Abstract

Diabetes mellitus affected more than 500 million of people globally, with an annual mortality of 1.5 million directly attributable to diabetic complications. Oxidative stress, in particularly in post-prandial state, plays a vital role in the pathogenesis of the diabetic complications. However, oxidative status marker is generally poorly characterized and their mechanisms of action are not well understood. In this work, we proposed a new framework for deep characterization of oxidative stress in erythrocytes (and in urine) using home-built micro-scale NMR system. The dynamic of post-prandial oxidative status (against a wide variety of nutritional load) in individual was assessed based on the proposed oxidative status of the red blood cells, with respect to the traditional risk-factors such as urinary isoprostane, reveals new insights into our understanding of diabetes. This new method can be potentially important in drafting guidelines for sub-stratification of diabetes mellitus for clinical care and management.

## Introduction

Diabetes mellitus (DM) is a multi-factorial metabolic disease which involve multiple genes and environmental factors, as well as other metabolic disorders such as obesity and insulin resistance, which is estimated to affect 578 million people worldwide by 2030^1,2^. Metabolic diseases are often present for years before clinical manifestation. In the long-term, the lesions in both the macrovascular system and microvascular system are the main reason of morbidity and mortality in diabetes patients, brings enormous economic and public health burdens^3,4^. Current clinical and laboratory predictors (e.g., body mass index, fasting glucose, and HbA_1c_) can serve as indicators to gauge diabetes risk, however, provide little insight into the aetiology and disease pathogenesis^5,6^.

Oxidative stress plays a vital role in the pathogenesis of the diabetic complications; however, oxidative stress is poorly characterized and their mechanisms of action are not well understood. The postprandial state is associated with physiological changes that occur during the interval between the meal consumption and the return of plasma glucose, amino acids, and triglycerides to pre-meal levels^7^. In particularly, the episode of post-prandial glucose spikes occurs due to an imbalance between glucose intake and the body’s ability to utilize or store glucose effectively. Hyperglycaemia and glucose spikes in individuals with Type-2 DM (T2DM) contribute to increased oxidative stress.

The damage to the macrovascular system which manifested as cardiovascular disease, is the primary cause of the mortality associated with diabetes^8^. Meanwhile, the more prevalent harm on the microvascular system in the retinopathy, neuropathy, and nephropathy also makes up a portion of the mortality^9,10^. Laboratory tests assessing indicators of oxidative stress are conventionally performed in a fasting state^11^, yet the postprandial state constitutes a significant proportion of the day, and consequently measurements obtained during the fasting state may not accurately depict the entire spectrum of oxidative stress within the body.

Currently, biomarker detection is the primary method for investigating postprandial oxidative stress by liquid biopsy (e.g., blood, plasma, urine) (details are shown in Table 1). Isoprostane (IsoP) is considered as a gold standard marker of in vivo oxidative stress and also a measure of environmental redox status by inducing inflammation and atherosclerosis through activation of mitogen-activated protein (MAP) kinase in many human diseases, i.e., periodontitis disease and chronic kidney disease^12–16^. IsoP concentrations in urine, however, may not truly and completely reflect systemic IsoP due to rapid clearance and production from the kidney^17^. Oxidative stress induces the oxidation of hemoglobin and damage to erythrocyte membranes^18^. Therefore, the evaluation of redox properties in erythrocytes provides valuable insights into the functional phenotyping of various biological pathways, thereby enhancing our understanding of the pathophysiology associated with the disease^19^.

**Table 1:** Translational clinical measurements of oxidative stress in reported case studies^61–73^

Liquid biopsy	Oxidative stress biomarker	Study design	Methodology	Cohorts	Reference
Blood	DNA damaged in blood leukocytes	Randomized controlled, clinical trial	Act diff 2 hematology analyzer/agilent scanner (G2505C)	47 female subjects (26 pregnant,21 non-pregnant)	Jiang^62^
Blood	Nitrite and nitrate	Randomized, interventional study	Relevant kit	24 white rabbits	Madihi^63^
Blood	RBCs	Targeted metabolomics approach	LC/MS analysis	Five male and three female	Fu (2016)
Blood	Mononuclear cells: Keap-1 protein levels and NQO1 protein levels, and nuclear Nrf-2 DNA binding capacity	Placebo-controlled, crossover intervention study	Not reported	10 healthy male subjects (aged 37, BMI 22.6)	Ghanim^64^
Plasma	Plasma	Crossover, acute interventional study	spark® multimode microplate reader	14 healthy subjects (aged 20–30)	Papagianni^65^
Plasma	Plasma, MDA	Randomized, controlled crossover trial	Not reported	11 healthy male subjects (aged 25.0, BMI 24.7)	Chusak^66^
Plasma	OxLDL	Randomized, double-blind, parallel-groups, placebo-controlled study	Oxidized LDL ELISA kit	146 healthy normal weight subjects (66 males, 80 females)	Deplanque^67^
Plasma	8 genes related to oxidative stress: NM_697; NM_2985; NM_14080; NM_1498; NM_581; NM_2574; mithocondrial proton carrier; NM_3355	Randomized crossover, clinical study	Specific RT2 Profiler PCR Arrays	22 healthy subjects	De Lorenzo^68^
Plasma	PGF2α	Double-blinded, randomized, placebo-controlled trial	Enzyme-linked immunosorbent assay kits	51 T2DM patients	Wu^69^
Serum	SOD	Clinical trial	Colorimetric platereader	17 male subjects (aged 18–35)	Hunter (2023)
Serum	ROS	Clinical interventional trial	Highly sensitive fluorescent probe	12 healthy male subjects	Laura^70^
Urine and blood	8-Hydroxydeoxyguanosine (8-OHdG), isoprostane and hs-CRP	Clinical trial	Enzyme-linked immunosorbent assay kit/enzyme-linked immunosorbent assay kits	10 T2DM patients (aged 33.9)	Kakuda^71^
Urine	MDA, SOD, CAT, GPx, GSH,GST	Randomized, double-blind, placebo-controlled trial	Enzyme-linked immunosorbent assay (ELISA) kits	90 healthy subjects	Lee^72^

**Fig. 1:**
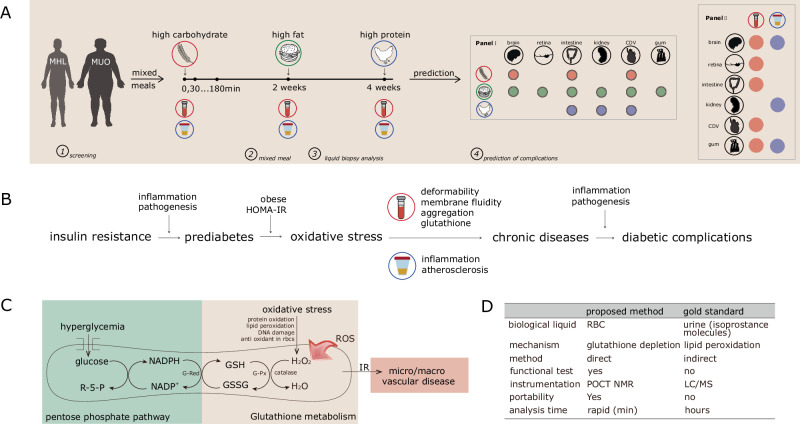
Nutritional load in post-prandial oxidative stress and the pathogeneses of diabetes mellitus. **A** The study design (outline) for the impact of mixed meal challenges with respect to post-prandial oxidative stress in metabolic healthy lean (MHL) and metabolic unhealthy obese (MUO) subjects. Aliquots of blood samples were withdrawn at every 30 min interval while urine samples were collected at beginning (0 min) and 360 min after mixed meal challenge (i.e., HC, HP, and HF) from both subgroups. We hypothesized that the mixed meal challenges is predictive of diabetic complications (Panel 1) via the deep phenotyping of oxidative stress in urine and erythrocytes samples (Panel 2)^44,52,61,73–84^. **B** Pathways from the onset of diabetes mellitus towards the development of diabetic complications. **C** The persistent condition of hyperglycaemia increases the production of H_2_O_2_, which is the first step of glycolysis in pentose phosphate pathway (PPP). The high endogenous rate of H_2_O_2_ production from hemoglobin autoxidation induced reduces the concentration of GSH and increases the oxidized glutathione (GSSG) levels, which leads to micro and macro vascular complications. **D** The salient features between the proposed NMR-based methods (oxidative status in erythrocytes) against the gold standard, urinary F_2_-IsoP. Adapted under the terms of the CC BY 4.0 licence^19^.

In this work, we proposed a non-invasive technique that allows rapid quantification of oxidative status in erythrocytes using micro-scale NMR^20–24^. The dynamic of post-prandial oxidative status and glucose spikes were studied extensively marked against the gold-standard, urinary-Isoprostane molecules. We hypothesized that mixed meal challenges are predictive of diabetic complications via deep phenotyping of post-prandial oxidative stress in erythrocytes and in urine samples (Fig. 1A, Table 2, and Supplementary Tables 1–5). Glutathione (GSH), a major intracellular antioxidant plays a key role in reducing the effects of oxidative stress (Fig. 1B–D). We observed that the oxidative stress bi-plot analysis can be used to stratify diabetic subjects into subgroups predictive of their diabetic complication^25,26^.

**Table 2:** Reported diabetic complications prediction based on the mixed meals study^44,52,61,73–83^

Organ/tissue	Cohorts	HF	HC	HP	Meals	Reference
Cardiovascular	7 subjects with Alzheimer’s disease and 9 cognitively normal control	●			40% saturated fat breakfast meal	Altman^74^
	17 males (aged 18–35)	●			Vanilla milkshakes (contain vanilla ice cream, heavy whipping cream, and whole milk)	Hunter (2023)
	Review paper	●			Not related	Uribarri^82^
	Review paper	●	●		Cream; refined carbohydrates	Biobaku^44^
	Rats	●		●	Lean chicken and beef	Hecke^77^
Retina	30 individuals diagnosed with type 2 diabetes without diabetic retinopathy (aged 54.6 ± 10.4)	●			Not related	Hamayel^76^
	Volunteers (aged 25–45)	●			Cow’s milk cream	Montserrat-de la Paz^80^
Kidney	Female Kunming mice weighing ~25 g (4 weeks old)			●	Not related	Yang^83^
	Male Sprague-Dawley rats (150 g)			●	Lean chicken and beef	Jakobsen^84^
	Rats	●		●	Lean chicken and beef; fat chicken and beef	Hecke^77^
	Simulated gastric fluid	●			Peroxidized food	Kanner^78^
Brain	Rats	●			Fat chicken and beef	Hecke^77^
	19 young males (aged 51 ± 6, BMI 23 + 4), 19 aged males (aged 67 ± 5, BMI of 27 ± 3)	●			Heavy whipping cream, syrup, sugar, milk	Marley^79^
	Review paper	●			Not related	Uribarri^82^
	Adult male Wistar rats weighing 200–250 g	●	●		Not related	Alzoubi^75^
Intestine	female mice weighing 25 g (4 weeks old)			●	Not related	Yang^83^
	Male Sprague-Dawley rats (250–300 g)	●			4% peroxidized menhaden oil chow	Tsunada^81^
	Rats	●	●		Not related	Lasker^52^
Gum	Mice	●			Not related	Zhou^61^

## Results

### Post-prandial oxidative stress in erythrocytes

**Fig. 2:**
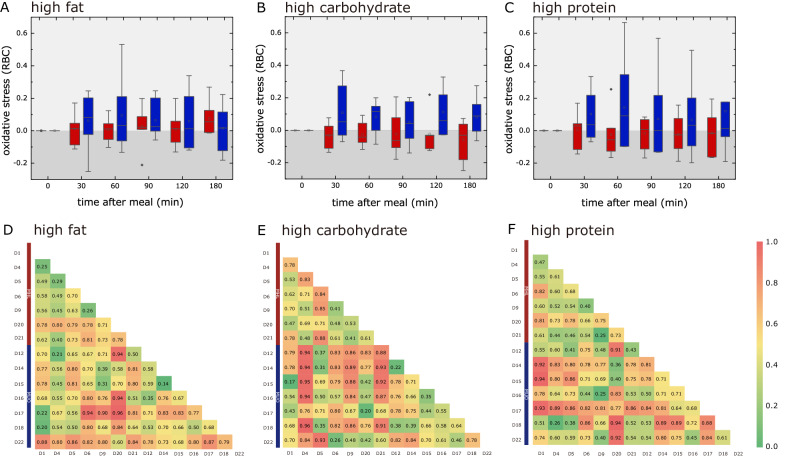
Post-prandial oxidative stress in erythrocytes. The oxidative status in erythrocytes of obese insulin-resistant subjects (blue, *n* = 7), and lean insulin-sensitive subjects (red, *n* = 7) challenged with mixed meals, **A** high fat meal, **B** high carbohydrates, and **C** high protein meal. The differentiation between MHL subgroup and MUO subgroup responses to the mixed meal challenges were calculated using receiver operating characteristic analysis. Hierarchical clustering of oxidative stress in erythrocytes levels depicting correlation among 14 subjects in **D** HF meal, **E** HC meal, and **F** HP meal with AUCs evaluated using ROC analysis represented in the form of heatmap. Open-labeled, randomized, cross meal intervention trial was carried out on (i) 7 MUO (27.5 < BMI < 35 kg/m^2^) insulin-resistant (HOMA-IR > 2.5) subjects and (ii) 7 MHL (20 < BMI < 23 kg/m^2^) insulin-sensitive (HOMA-IR < 1.6) subjects. High fat, high protein, and high carbohydrates meal were challenged once every 2 weeks, and aliquots of blood were withdrawn at every 30 min interval for 2 h. Redox phenotyping of the erythrocytes was measured by using micro-scale NMR system. Other traditional markers (fasting glucose, insulin, cholesterol etc.) were also recorded.

The MUO subgroup (blue) exhibited relatively higher (than MHL subgroup (red)) postprandial oxidative stress for all meal challenges, in particularly the HF meal and HC meal challenges (Fig. 2A–C). This is expected as the glycemic index (GI) and glycemic load (GL) were relatively much higher in HF meals and HC meals than its’ counterpart (HP). In contrast, the MHL group demonstrated less profound oxidative stress in all the meal challenges (*P* < 0.05), with only the minor exception of HF meal where the levels of oxidative stress were slightly elevated. The HC meal (followed by HP, and HF) provides the relatively the largest contrast between each subgroup (Fig. 2D–F). An increase in glucose may lead to the promotion of inflammation through the activation of cell signaling pathways mediated by NF-κ band aggravating oxidative stress^27,28^. In contrast, slight antioxidants were generated in the erythrocytes of individuals in MHL subgroup under the HC meal and HP meal challenges. The differences in oxidative status between subjects in the MHL group were less profound (*P* > 0.05).

### Post-prandial oxidative stress in urine

**Fig. 3:**
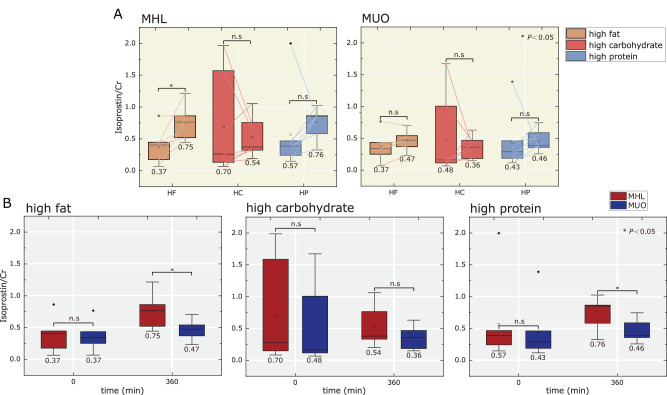
Post-prandial oxidative stress in urine. The oxidative stress in urinary-IsoP for **A** MHL subgroup (red, *n* = 7) and MUO subgroup (blue, *n* = 7), **B** challenged with mixed meals (i.e., high fat, high carbohydrate, and high protein), where the before meal (0 min) and after meal (360 min) readings were recorded.

The oxidative stress in urinary (Isoprostane molecules) were marked elevated in all the meal challenges (except for HC meals), in both the MHL subgroup (0.37 to 0.75, 0.57 to 0.76) and MUO subgroup (0.37 to 0.47, 0.43 to 0.46). The values in the parentheses were for the HF meals and HP meals, respectively (Fig. 3A). Similar trends were also observed in HF meals and HP meal have also been reported by Kurti et al.^29^, and Mok et al.^30^, respectively. For HC meals, the unexpected decreasing trends of both the subgroups were due to two subjects with abnormal high urinary-Isop at the beginning diverging from the increasing tread showed by five other subjects in each subgroup. This ‘anomaly’ may change with increasing number of participating subjects in the study. McAnulty et al.^31^ postulated that this was probably attributed to the reduction of cortisol and epinephrine hormone levels after the ingestion of HC-based beverages. The difference in oxidative stress in urine was not significant (*P* > 0.05) prior to meal between MHL subgroup (0.37, 0.70, 0.57) and MUO subgroup (0.37, 0.48, 0.43) for HF meals, HC meals, and HP meals (Fig. 3B). We found that there were two individuals who had high levels of oxidative stress in urine at 0 min in both subgroups, respectively. Oxidative stress in urine in MHL subgroup (HF, HP) was much higher than MUO subgroup (HF, HP) at 360 mins after the meals (*P* < 0.05) (Fig. 3B).

### Sub-stratification of diabetic mellitus—integrated analysis between oxidative stress in urine and erythrocytes

**Fig. 4:**
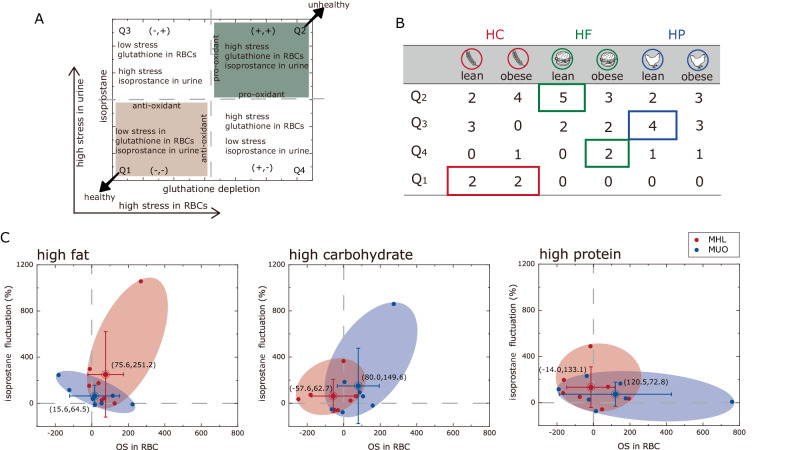
Bi-plot analysis of oxidative stress in urine and erythrocytes for sub-stratification diabetes mellitus. **A** Quadrant plot illustrating oxidative stress in erythrocytes (in X-axis) and the orthogonal coordinate representing the Isoprostane fluctuations (in Y-axis). The pro-oxidative stress and antioxidant quadrant are Q1 and Q4, respectively. **B** the number of individuals from the MHL subgroup(red) and MUO subgroup (blue) after the three mixed meal challenges, (**C**) oxidative stress bi-plot visualizing the relation between MHL subgroup and MUO subgroup under three meal challenges (i.e., high fat meal, high carbohydrates, and high protein meal).

The urine and blood samples were taken from all the subjects after the meal challenges, and the changes in urinary-IsoP and oxidative status in erythrocytes were measured (Fig. 4A, B). Overall, the IsoP in urines were elevated while the oxidative status in erythrocytes varies depending on the individuals. This meal challenge shows a well-distributed balance between pro-oxidative and anti-oxidative in the MHL subgroup (*P* > 0.05). As much as five subjects having elevated risks were interestingly, recorded in Q4. This would otherwise be not possible without using the proposed methods (or using single traditional marker).

We found that a unique subject whose urinary-IsoP was significantly higher than others within the MHL group after HF meal. Expectedly, this individual has the highest HOMA-IR within the MHL subgroup. Similar finding which observed positive correlation between HOMA-IR and urinary-IsoP levels was reported^32^. Another interesting finding is the abnormally high post-prandial oxidative stress for individuals in MUO group after the HP meal challenges (Fig. 4C). Expectedly, this subject also has the highest systolic and diastolic blood pressure within the MUO subgroup. Bonifácio et al. studies also suggesting that nitro-oxidative stress was significantly associated with the increased of blood pressure^33^.

### Dual markers—integrated blood glucose–oxidative stress analysis

**Fig. 5:**
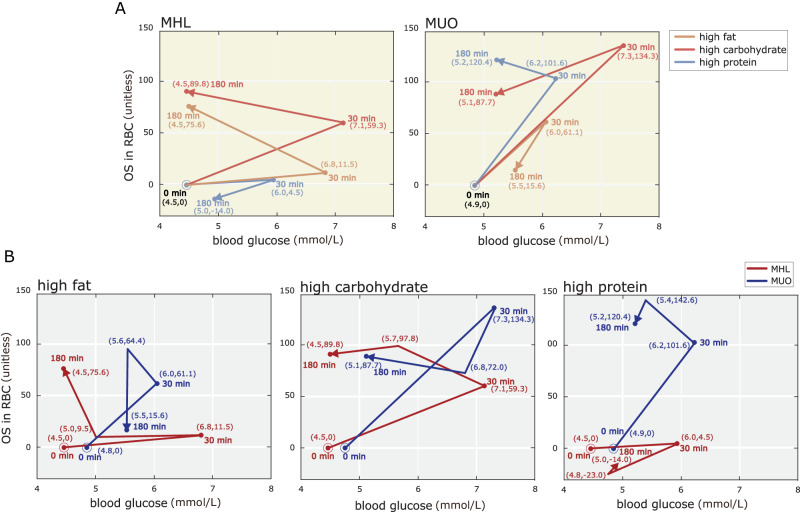
Integrated blood glucose and oxidative stress analysis. Blood glucose and oxidative stress bi-plot analysis for (**A**) between MHL subgroup (red, *n* = 7) and MUO subgroup (blue, *n* = 7), and according to (**B**) meal challenges (i.e., high fat meal, high carbohydrates, and high protein meal). Each data point was collected at 30 min intervals ranging from 0 min (circle) to 180 min.

The glucose after spikes were observed (30 min) in (HF, HC, HP) meals were (6.8, 7.1, 6.0) in MHL subgroup and (6.0, 7.3, 6.2) in MUO subgroup, respectively (Fig. 5A). Interestingly, the glucose levels gradually returned to the baseline by (around) 180 min. We noticed subjects in MHL subgroup were able to return much faster than MUO subgroup. Similarly, the level of oxidative stress in erythrocytes increased during the glucose spikes intervals (30 min). However, while the glucose had returned to baseline the oxidative stress levels remained elevated (30 min to 180 min). The spikes for HC meals in both the (MHL, MUO) subgroups are the highest (7.1, 7.3), while that for HP meal is the lowest (6.0, 6.2). This can be attributed to the higher GI in HC, which translate into a higher and direct absorbance of glucose in the gastrointestinal tract and a faster than the erythrocyte uptake rate^34^.

The stark contrast in antioxidant between MHL subgroup and MUO subgroup lies in that the glucose concentration of candidates in MHL subgroup rapidly returned to the baseline (4.5 mmol/L in three meals), in contrast to the MUO subgroup (5.2, 5.5, 5.2). On one hand, over-nutrition causes oxidative stress, which leads to an elevated insulin level; on the other hand, MUO subjects with high level of insulin resistance have impaired response to the insulin, resulting in a failure in reducing the blood glucose concentration in time^35^. Therefore, oxidative stress dual markers can potentially be a predictive marker for onset of diabetic mellitus (or even the development of diabetic complications). The dynamics of blood glucose and oxidative stress were in the similar fashion for both the MUO and MHL subgroup (HC meals) and stabilized after 180 min, which is characterized by the blood glucose. In contrast to the HP meal, the MUO subgroup and MHL subgroup seems to be heading towards the opposite direction. The trajectories for HF meals, both the metabolic subgroups were however, not well-defined (Fig. 5B).

## Discussion

In this work, we proposed a new framework for deep characterization of oxidative stress in erythrocytes using home-made micro-scale NMR to provide a rapid, cost-effective, and easy-operating alternative. This study presents among the first exploration of post-prandial oxidative stress in erythrocytes (with benchmarking against the standard urine-Isop) from mixed meals as a mean to predict diabetes and its complications. The assessment of redox properties in erythrocytes can provide valuable parameters for functional phenotyping of various biological pathways, facilitating the insight into diabetes pathophysiology^19^. RBC does not have mitochondria and may not reflect oxidative stress found in cells with mitochondria^36–38^. Nonetheless, we believe that this may provide information on the ‘ambient’ oxidative stress of the blood that is less affected by cellular response other stimuli (due to the lack of mitochondria)^39,40^. Existing blood biomarkers such as glycated hemoglobin are effective in predicting diabetic complications, e.g., microangiopathy and retinopathy^41,42^. In parallel, urinary IsoP are (indirectly) modified by a series of downstream events that indicate changes in the metabolism of IsoP^43^. The impact of meal on diabetic complications can be reflected in postprandial oxidative stress^44^, which is an area requiring systematic exploration for further optimize diabetes care.

The platform used in this study is compact and suited for point-of-care testing. These findings hold promises for clinical applications in complimentary to existing clinical care and diabetes management. Direct quantitative analysis of ROS is complex due to their highly reactive nature and short half-lives. Therefore, the level of oxidative stress is primarily assessed indirectly by measuring the markers of oxidative damage rather than the oxidative species themselves^45^. Biomarkers resulting from lipid peroxidation or GSH depletion (Table 2) can be generated and evaluated using conventional biochemical techniques, which are costly and require intricate purification procedures, advanced instrumentation, and substantial effort^46^.

Our results showed a higher degree of oxidative stress and abnormal blood glucose levels in the MUO group, which may be associated with meal-induced oxidative damage to critical proteins involved in glycolysis, the TCA cycle, and ATP synthase, ultimately leading to impaired glucose metabolism^47^. Notably, subject-10 in MUO subgroup exhibited the most significant oxidative stress in erythrocytes under HP meal (Supplementary Table 3), and displayed the highest HOMA-IR. Previous studies have consistently reported a direct positive correlation between HOMA-IR and oxidative stress^35,48^. In obese individuals, elevated insulin levels in the fasting state and following protein intake may trigger oxidative stress-induced insulin resistance mediated by adipocyte-derived factors, including TNF-α, leptin, and free fatty acids, which are early indicators of diabetes^49^.

One of the major limitations of this study, however, there is lack of evidence of direct linkage of the proposed biomarkers with complications of diabetes (e.g., longitudinal follow-up study), which is important evidence base towards clinical utility of the proposed biomarkers. This is beyond the scope of our current (proof-of-concept) study, which is focused on the assessment of oxidative stress using red blood cell and urine biomarkers following meal interventions. We nonetheless, acknowledged the importance of linking the biomarker with diabetes complications as a limitation of our study and future research direction.

In summary, we demonstrated that deep phenotyping of oxidative stress in erythrocytes could be a novel biomarker that (indirectly) reflects the GSH depletion, which can be detected non-invasively through novel micro-scale NMR technology developed in this work. The proposed integrated dual-markers proposed in this study reveals unique relationship in oxidative stress of erythrocytes and urine, and secondly, we demonstrated showed a potential correlation between blood glucose concentration and oxidative stress in erythrocytes. Substantial new insights into the impact of meal modifications on the oxidative stress were revealed in this study, which would be impossible without the micro-NMR platform.

## Methods

This study was designed and conducted according to the Singapore Good Clinical Practice Guideline and principles of the 2013 Declaration of Helsinki. Singapore’s National Healthcare Group Domain Specific Review Board (DSRB Ref No: C/2013/00902) reviewed and approved the protocol of this study. All subjects provided written consent before participation in this study.

### Subjects and study design

Fourteen Chinese men aged 21–40 years old were recruited into this duty and divided into two groups according to their BMI and insulin resistance, in detail, one group consists of seven metabolically unhealthy obese (MUO) (BMI ≥ 27.5 kg/m^2^) that are insulin-resistant (HOMA-IR 4.34 ± 0.41), while the other group consists of seven metabolically healthy lean (MHL) (18.5 kg/m^2^ ≤ BMI ≤ 23 kg/m^2^) who are insulin-sensitive (HOMA-IR 0.83 ± 0.10). It is worth mention that Asians with BMI over 27.5 are classified into obesity, as Asians have higher risk of metabolic disease^50^. Height, weight, and waist circumference were measured; fasting blood glucose level was also assessed for the determination of plasma glucose, serum insulin, electrolytes, non-esterified fatty acid (NEFA) concentrations, and lipid profile. Body weight, measured with lightweight clothing, was recorded to the nearest 0.1 kg using an electronic scale (HN-289, OMRON, Japan). Height was measured barefoot, to the nearest 0.1 cm, utilizing a wall-mounted stadiometer. Body mass index (BMI) was calculated by dividing weight in kg by their square of height in meters. Waist circumference (WC) was evaluated with participants in erect standing position and relaxed state. Plasma glucose and triglyceride (TG) concentrations were measured by enzymatic and colorimetric methods (AU5800, Beckman Coulter Inc., California, USA). Serum insulin was measured using a chemiluminescence immunoassay (ADVIA Centaur, Siemens Healthcare Diagnostics, Hamburg, Germany). These testing criteria and analytical methods were carried out by a laboratory accredited by the College of American Pathologists. Insulin-sensitive lean subjects (MHL) were identified by a Homeostatic Model Assessment-Insulin Resistance (HOMA-IR), with scores below 1.2, while insulin-resistant obese subjects (MUO) were identified by a HOMA-IR, with scores equal or greater than 2.5 (details are shown in Table 3).

**Table 3:** Characteristics of study participants in this study

	MHL (*n* = 7)	MUO (*n* = 7)	r	*P*-value
age (years)	23.3 ± 0.3	28.6 ± 1.2	0.778	0.001
BMI (kg/m^2^)	21.9 ± 0.2	30.6 ± 0.9	0.868	<0.001
weight (kg)	66.6 ± 2.3	87.1 ± 3.9	0.834	<0.001
height (cm)	174.1 ± 3.2	168.7 ± 3.4	−0.302	0.295
WC (cm)	79.7 ± 0.7	101.0 ± 1.2	0.869	<0.001
SBP (mmHg)	110.0 ± 4.4	120.0 ± 2.6	0.621	0.018
DBP (mmHg)	58.4 ± 2.7	72.9 ± 3.6	0.675	0.008
Chol (mmol/L)	5.1 ± 0.3	5.5 ± 0.5	0.124	0.673
TG (mmol/L)	0.6 ± 0.1	1.8 ± 0.2	0.727	0.003
HDL-c (mmol/L)	1.7 ± 0.1	1.3 ± 0.1	−0.797	0.001
LDL-c (mmol/L)	3.0 ± 0.4	3.4 ± 0.4	0.053	0.857
insulin/Scr (mmol/L)	4.8 ± 0.5	19.3 ± 1.6	0.868	<0.001
FBG/Scr (mmol/L)	4.3 ± 0.1	4.7 ± 0.1	0.732	<0.001
HOMA-IR	0.9 ± 0.1	4.1 ± 0.4	0.868	<0.001

The baseline characteristics were established after 10 h of overnight fasting.

### Study protocol

The eligible subjects underwent a dietary tolerance test of three different macronutrient compositions of isocaloric meal, namely, high fat (HF), high carbohydrate (HC), and high protein(HP), in a randomized order with a 7-day washout period between each trial. Informed consent was secured from all participants enrolled in this study. The anticipated inflammatory reactions in response to a high-fat meal are likely to be more intricate and nuanced than previously comprehended^51^. The HF meal has been demonstrated to elicit pro-inflammatory and oxidative stress responses^52,53^. Depending on an individual’s metabolic status and the fatty acid composition in the test meal, these reactions may vary. Consequently, it is imperative to elucidate these inconsistencies by investigating the impact of a standardized high-fat meal, comprising equivalent proportions of polyunsaturated (PUFA), monounsaturated (MUFA), and saturated fatty acids (SFA). Regular consumption of the HC meal elicits a notable post-meal surge in oxidative stress, blood glucose levels, and inflammatory reactions^54^. When this pattern recurs throughout the day, it creates an environment conducive to the advancement of atherosclerosis and the onset of cardiovascular disease^55^. In contrast, the HP meal exhibits a lower glycemic index^56^. However, findings from meta-analyses suggest a potential link between elevated consumption of animal protein and an increased risk of developing T2DM^57^. Furthermore, data from numerous prospective studies indicate that animal protein, a significant component of various ketogenic diets, may elevate the risk of chronic kidney disease (CKD)^58,59^. HF, HC, and HP meals contained 56.5% fat (with a 1:1:1 ratio of SFA, MUFA, and PUFA), 56.4% carbohydrate, and 51.4% protein, respectively (detail in Supplementary Table 6). Furthermore, to mitigate the influence arising from energy disparities, their overall energy content was essentially equated in the final analysis (~600 kJ).

### Selection criteria

Exclusion criteria for the subjects include current smoking, previous or current thyroid disorder, history of malignancy, hospitalization or surgery within the past 6 months, intervention for dyslipidaemia, use of corticosteroids within the past 3 months, alcohol consumption >3 units daily, moderate to high-intensity physical activity >5 h per week, weight change ≥5% within the past 3 months, and a first-degree relative with Type 2 diabetes.

### Biochemical analysis

#### Blood sample analysis

Fasting and postprandial (0, 30, 60, 90, 120, 180 min) venous blood samples from the subjects were collected into plastic tubes containing EDTA-2Na (VACUETTE1, Greiner Bio-One, Austria) and separated into two parts: one for novel micro-scale NMR measurement^19^ and the other for glucose concentration measurement.

### Oxidative status measurement with micro-scale NMR

Freshly collected erythrocytes were incubated and oxidized with 6 mM sodium nitrite in phosphate-buffered saline (PBS) in a 1:1:8 (w/v) ratio for 10 min, washed three times by PBS to stop the reaction and re-suspended in PBS. All blood samples were either utilized immediately or stored at 2 °C, and used within four days (unless otherwise specified) after collection. For micro-scale NMR measurements, the microcapillary tubes (Fisher Scientific, PA) were employed to transfer the processed blood, followed by centrifugation (6000 × *g* for 1 min) to obtain the packed erythrocytes and R_2_ (relaxation rate) was obtained by micro-scale NMR. The actual amounts of nitrosative stress (‘normalized’) were calculated by subtracting the baseline (0 min).

Plasma glucose concentration was determined using enzymatic methods (AU5800, Beckman Coulter Inc., California, USA).

#### Urine sample analysis

Fasting and postprandial (0, 360 min) urine samples from the subjects were collected and promptly frozen before being sent for urinary F2-IsoP analysis using LC/MS. Urinary free F2-IsoPs were processed via anionic solid-phase extraction. Creatinine levels were measured to standardize urine dilution using a Photometric Analyzer (Roche Diagnostic GmbH, Germany). Subsequently, samples were analyzed using gas chromatography–mass spectrometry, operating in negative chemical ionization mode (Agilent Technologies, CA), with a Triple-Axis Detector, connected to a gas chromatograph (Agilent Technologies, CA). Quantification was accomplished by comparing the peak area of free F2-Isoprostanes with that of the corresponding deuterated internal standard.

### Data analysis

All statistical analyses were performed using SPSS of version 23.0 (SPSS Inc., Chicago, IL, USA). All values are presented as means ± standard errors (SEMs). Student’s t-test was performed to examine the associations between postprandial immune metabolism parameters between and within the subgroups. A *P*-value < 0.05 was considered statistically significant.

### Receiving operating characteristic

The analyses were employed to assess the specificity and sensitivity of the diagnostic techniques. Various supervised models, including kNN, Logistic Regression, Naïve Bayes, Neural Network, and Random Forest, were utilized for the ROC tests^60^. A power function fitting the form y = ax^b^ was applied throughout the study, with iterations executed using the Levenberg–Marquardt algorithm until a chi-squared tolerance of 10^−9^ was attained. The postprandial oxidative stress result (combine 0, 30, 60, 90, 120, 180 min) for each subject following each meal (HC, HF, HP) was determined using micro-scale NMR and utilized for calculating Area Under the Curve (AUC). The resulting function’s AUC was then compared to the actual averaged AUC derived from all the supervised models.

### Supplementary information

Supplementary Information

## Data Availability

The primary data can be found in Supplementary Information, and referenced datasets (datasets analyzed in the study) is available upon request at pengwengkung@sslab.org.cn.
