# Author Correction: Nutritional load in post-prandial oxidative stress and the pathogeneses of diabetes mellitus

**DOI:** 10.1038/s41538-024-00288-5

**Published:** 2024-07-16

**Authors:** Fangzhou He, Junshi Liu, Yuanding Huang, Lan Chen, Ehsan Parvaresh Rizi, Ke Zhang, Lijing Ke, Tze Ping Loh, Meng Niu, Weng Kung Peng

**Affiliations:** 1 Songshan Lake Materials Laboratory, Dongguan, China; 2 Dongguan Institute of Technology, Dongguan, China; 3 BioSyM, SMART Centre, Singapore, Singapore; 4 National University of Singapore, Singapore, Singapore; 5 School of Food Science and Nutrition, University of Leeds, Leeds, UK; 6 National University of Health System, Singapore, Singapore; 7 Department of Interventional Radiology, Shengjing Hospital of China Medical University, Shenyang, China

**Keywords:** Diabetes complications, Nutrition, Pre-diabetes

Correction to: *npj Science of Food* 10.1038/s41538-024-00282-x, published online 27 June 2024

In this article the author name Ke Zhang was incorrectly written as Zhang Ke. The ORCiD for Fangzhou He was incorrect and should have been 0009-0007-3154-4376. The original article has been corrected.

