# Supplementary material for: Nutritional load in post-prandial oxidative stress and the pathogeneses of diabetes mellitus
Source: NPJ Sci Food. 2024 Jun 27;8:41. doi: 10.1038/s41538-024-00282-x (PMC11211471; doi:10.1038/s41538-024-00282-x)
Supplement: Supplementary file 1 — Supplementary Information [file 41538_2024_282_MOESM1_ESM.pdf]

Supplementary Information

Nutritional load in post-prandial oxidative stress and the pathogeneses of Diabetes Mellitus

Fangzhou He[1], Junshi Liu[2], Yuanding Huang[1], Lan Chen[3], Ehsan Parvaresh Rizi[4], Ke Zhang[1], Lijing Ke[5],Tze Ping Loh[6], Meng Niu[7]*, Weng Kung Peng[1,3]*

[1]Songshan Lake Materials Laboratory, Dongguan, 523-808 China.

[2]Dongguan Institute of Technology, Dongguan, China.

[3]BioSyM, SMART Centre, Singapore.

[4]National University of Singapore, Singapore.

[5]School of Food Science and Nutrition, University of Leeds, United Kingdom

[6]National University of Health System, Singapore.

[7]China Medical University, Shenyang, China.

	Isoprostane	MHL (n=7)	MUO (n=7)	*P*-value
HF	0 min	0.37±0.24	0.36±0.19	0.993
	360 min	0.75±0.24	0.47±0.14	0.028
	0 min/ 360 min (ratio,%)	251±342	66±85	0.220
HC	0 min	0.70±0.71	0.48±0.57	0.556
	360 min	0.54±0.28	0.36±0.15	0.199
	0 min/ 360 min (ratio,%)	63±133	150±302	0.531
HP	0 min	0.57±0.59	0.43±0.40	0.653
	360 min	0.76±0.22	0.46±0.15	0.015
	0 min/ 360 min (ratio,%)	133±163	73±96	0.450

Supplementary Table 1.Average changes in Isoprostane at 0 and 360 min in MHL and MUO groups under mixed meal challenges. Data presented as mean ± standard errors of mean

Mixed meal	group	0	30	60	90	120	180
HF	MHL	0	0.0115	0.0056	0.0270	0.0095	0.0756
	MUO	0	0.0611	0.0945	0.0644	0.0594	0.0156
HC	MHL	0	0.059333	0.054484	0.097781	0.069289	0.089832
	MUO	0	0.134264	0.072019	0.100993	0.114428	0.087734
HP	MHL	0	0.00448	-0.02464	-0.01578	-0.02291	-0.014
	MUO	0	0.101553	0.142592	0.072552	0.066307	0.120449

Supplementary Table 2.Average changes in NMR signal at 0-180 min in MHL and MUO groups under mixed meal challenges

Supplementary Table3. Recruited subjects' physical detail

No.	W	H	BMI	group	Age	SBP	DBP	WC	Chol	TG	HDL-c	LDL-c	Insulin/Scr	FBG/Scr	HOMA-IR
1	59	163	22.21	LEAN	23	135	65	80	7.1	0.61	1.64	5.18	5.26	4.5	1.05
2	64.4	172.5	21.64	LEAN	23	110	55	82	4.63	0.48	1.75	2.66	6	4.3	1.15
3	73.5	179	22.94	LEAN	25	107	52	78	4.87	0.92	1.76	2.69	4.2	4.1	0.77
4	59.3	163	22.32	LEAN	23	109	58	77	4.71	0.35	2.28	2.27	3.3	4.2	0.62
5	71.6	183	21.38	LEAN	23	104	61	81	4.66	0.55	1.62	2.79	5.9	4.3	1.13
6	66.6	175	21.75	LEAN	23	106	69	79	5.08	0.94	1.79	2.86	5.8	4.6	1.19
7	71.8	183.3	21.37	LEAN	23	99	49	81	4.38	0.55	1.35	2.78	3.1	4.2	0.58
8	73.2	151	32.10	OBESE	31	120	76	103	7.09	1.86	1.29	4.95	14.96	4.7	3.12
9	87.9	178	27.74	OBESE	27	114	61	96	4.14	0.88	1.36	2.38	18.72	4.7	3.91
10	84.2	164	31.31	OBESE	30	125	83	102	6	2.2	1.12	3.88	22.48	5.4	5.40
11	106.2	176	34.28	OBESE	30	111	63	106	4.92	2.02	1.27	2.73	14.24	4.4	2.78
12	90.5	171.5	30.77	OBESE	32	132	85	101	4.85	2.4	1.06	2.7	23.73	4.9	5.17
13	87.9	170	30.42	OBESE	27	120	76	100	4.25	0.9	1.28	2.56	24.09	4.4	4.71
14	80	170.5	27.52	OBESE	23	118	66	99	7.18	2.09	1.46	4.77	17.11	4.7	3.57

W:Weight (kg), H:height (cm), BMI: Body Mass Index (kg/m-2), SBP: systolic blood pressure (mmHg), DBP: diastolic blood pressure (mmHg), WC: waist circumference (cm), Chol: cholesterol (mmol/L), TG: Triglycerides (mmol/ L), HDL-C: High density lipoprotein cholesterol (mmol/ L), LDL-c: Low-Density Lipoprotein Cholesterol (mmol/ L), Scr: serum creatinine (mmol/ L), FBG: fasting blood glucose (mmol/ L).

group	ID	Isoprostane /Cr_0_HF	Isoprostane /Cr_360_HF	% Change Isoprotane/Cr
LEAN	MEAL01	0.40295594	0.520158272	29.08564451
	MEAL04	0.175626682	0.442972808	152.2240936
	MEAL05	0.440864451	1.215172862	175.6341231
	MEAL06	0.445460777	0.64879554	45.64594067
	MEAL09	0.861727397	0.862278185	0.063916625
	MEAL20	0.066053156	0.764535105	1057.454312
	MEAL21	0.201424359	0.802144293	298.2359911
OBESE	MEAL12	0.435541299	0.43739514	0.425640779
	MEAL14	0.326190668	0.54236676	66.27292362
	MEAL15	0.40195503	0.369853896	-7.986250186
	MEAL16	0.340023512	0.471563456	38.68554349
	MEAL17	0.067086364	0.231920396	245.7042281
	MEAL18	0.250435819	0.542019844	116.4306392
	MEAL22	0.765378434	0.704949556	-7.895294037

group	ID	Isoprostane /Cr_0_HP	Isoprostane /Cr_360_HP	% Change Isoprotane/Cr
LEAN	MEAL01	0.289604612	0.858773942	196.533241
	MEAL04	0.467063478	0.852396124	82.5011299
	MEAL05	0.386948236	0.581620637	50.30967517
	MEAL06	0.431350517	1.024174468	137.4343899
	MEAL09	1.997130632	0.842858139	-57.79654441
	MEAL20	0.24189467	0.324705952	34.23443834
	MEAL21	0.147070444	0.865836692	488.7224285
OBESE	MEAL12	0.117031393	0.387340865	230.9717644
	MEAL14	0.221640923	0.588440606	165.4927607
	MEAL15	0.187148598	0.256948943	37.29675028
	MEAL16	0.290597475	0.368066228	26.65843962
	MEAL17	0.462121637	0.498497843	7.871565259
	MEAL18	0.345593775	0.746245139	115.9313022
	MEAL22	1.389848415	0.356617762	-74.34124771

group	ID	Isoprostane /Cr_0_HC	Isoprostane /Cr_360_HC	% Change Isoprotane/Cr
LEAN	MEAL01	0.656490701	1.0616801	61.72050849
	MEAL04	0.192342858	0.331906661	72.55990934
	MEAL05	0.279514636	0.341753687	22.26683086
	MEAL06	0.081948899	0.381495876	365.52898486
	MEAL09	1.986596396	0.765579896	-61.46273607
	MEAL20	0.148983712	0.201642857	35.34557170
	MEAL21	1.587561013	0.675605577	-57.44380394
OBESE	MEAL12	0.065598608	0.629634401	859.82891108
	MEAL14	0.115349672	0.18471707	60.13662302
	MEAL15	0.13873171	0.394394055	184.28544124
	MEAL16	0.16291683	0.32	94.95002170
	MEAL17	1.673090739	0.361753998	-78.37810048
	MEAL18	0.187194661	0.149458398	-20.15883484
	MEAL22	1.006674626	0.468717442	-53.43903284

Supplementary Table 4.Changes in Isoprostane at 0 and 360 min in MHL and MUO groups under mixed meal challenges. Change is calculated by equation: Change (%) =((Isoprostane /Cr_360- Isoprostane /Cr_0)/ Isoprostane /Cr_0)*100

				HF			
group	ID	0	30	60	90	120	180
LEAN	MEAL01	0.0000	-0.1125	-0.1032	-0.2113	0.0125	0.0548
	MEAL04	0.0000	0.0132	0.0313	0.0872	-0.1310	-0.0141
	MEAL05	0.0000	-0.0874	-0.0536	0.0215	-0.0710	0.0367
	MEAL06	0.0000	0.0406	0.0435	0.0173	0.0589	0.0694
	MEAL09	0.0000	0.0458	0.0112	0.0673	0.0270	0.1251
	MEAL20	0.0000	0.1707	0.1235	0.1984	0.1991	0.2687
	MEAL21	0.0000	0.0103	-0.0134	0.0084	-0.0291	-0.0110
OBESE	MEAL12	0.0000	-0.0235	0.0322	-0.0023	0.0078	0.0537
	MEAL14	0.0000	0.0816	0.2103	0.0257	0.2109	0.1152
	MEAL15	0.0000	0.2018	0.0811	0.2013	0.0708	0.0169
	MEAL16	0.0000	-0.0125	0.0004	0.0308	0.0121	0.0052
	MEAL17	0.0000	-0.2514	-0.0626	-0.0572	-0.1056	-0.1818
	MEAL18	0.0000	0.1867	-0.1326	0.0065	-0.1194	-0.1231
	MEAL22	0.0000	0.2453	0.5326	0.2462	0.3392	0.2230

				HP			
group	ID	0	30	60	90	120	180
LEAN	MEAL01	0.0000	-0.14365	-0.16578	-0.02797	-0.02616	-0.1623
	MEAL04	0.0000	0.028305	0.014852	-0.16876	-0.18923	-0.16595
	MEAL05	0.0000	0.029012	-0.10119	-0.11336	-0.11023	-0.07502
	MEAL06	0.0000	0.02209	0.008184	0.04122	0.030539	0.07991
	MEAL09	0.0000	0.042228	-0.05652	0.006817	-0.097	0.04722
	MEAL20	0.0000	0.169227	0.254791	0.067957	0.157094	0.194694
	MEAL21	0.0000	-0.11585	-0.12679	0.083611	0.0746	-0.01652
OBESE	MEAL12	0.0000	-0.06827	-0.09845	-0.13099	0.032514	-0.03713
	MEAL14	0.0000	0.217997	0.14109	0.216451	0.089097	0.147303
	MEAL15	0.0000	0.211907	0.666068	0.000392	0.201055	0.176434
	MEAL16	0.0000	0.036904	0.09105	0.048754	-0.0621	-0.0255
	MEAL17	0.0000	0.33339	0.345024	0.569397	0.49538	0.759175

	MEAL18	0.0000	-0.04113	-0.09686	-0.13347	-0.19787	-0.19058
	MEAL22	0.0000	0.020078	-0.04979	-0.06267	-0.09392	0.013443

		HC					
group	ID	0	30	60	90	120	180
LEAN	MEAL01	0.0000	0.0250	0.0430	-0.0126	-0.0329	0.0724
	MEAL04	0.0000	-0.1110	-0.0746	-0.1791	-0.1136	-0.1812
	MEAL05	0.0000	0.0768	0.0922	0.2054	0.2191	0.0371
	MEAL06	0.0000	-0.0299	-0.0427	-0.0893	-0.0495	-0.0007
	MEAL09	0.0000	-0.1359	-0.0582	-0.1070	-0.0307	-0.0313
	MEAL20	0.0000	0.0041	-0.0219	0.0775	-0.0318	-0.2484
	MEAL21	0.0000	-0.0595	-0.1186	-0.0628	-0.1243	-0.0512
OBESE	MEAL12	0.0000	0.1512	0.1997	0.1763	0.3279	0.2745
	MEAL14	0.0001	0.2732	0.1179	0.2018	0.2185	0.1061
	MEAL15	0.0000	0.0522	0.0855	0.0411	-0.0270	0.0042
	MEAL16	0.0000	-0.0308	-0.0007	0.1258	0.0592	0.0875
	MEAL17	0.0000	-0.0701	0.1148	-0.1397	0.0445	-0.0057
	MEAL18	0.0000	0.3672	0.1479	-0.0038	0.1906	0.1591
	MEAL22	0.0000	0.0077	-0.0859	-0.0505	-0.0285	-0.0654

Supplementary Table 5.Changes in NMR readings at postprandial (0, 30, 60, 90, 120, 180 min) venous blood samples in MHL and MUO groups under mixed meal challenges.

		amount(g)	energy(Kcal)	carbohydrate (g)	protein (g)	fat (g)	MUFA (g)	PUFA(g)	SFA(g)
**HF meal**	Ensure Plus®	260.0	366.60	51.69	13.00	11.70	2.85	6.90	1.48
	butter	11.0	78.87	0.01	0.09	8.80	2.31	0.33	5.61
	diary cream heavy whipping	11.0	37.95	0.31	0.22	4.07	1.10	0.15	2.53
	peanut oil	13.0	114.92	0.00	0.00	13.00	5.98	4.16	2.18
	total		**598.34**	52.00	13.31	37.57	12.24	11.54	11.81
	% composition						32.6	30.7	31.4
	% kcal			34.8	8.9	**56.5**			
**HC meal**	Ensure Plus®	425.0	599.25	84.49	21.25	19.13	4.65	11.28	2.42
	total		**599.25**	84.49	21.25	19.13	4.65	11.28	2.42
	% composition						24.3	59.0	12.7
	% kcal			**56.4**	14.2	28.7			
**HP meal**	Ensure Plus®	230.0	324.30	45.72	11.50	10.35	2.52	6.11	1.31
	Beneprotein® Powder	77.0	274.89	0.00	65.45	0.00	0.00	0.00	0.00
	total		**599.19**	45.72	76.95	10.35	2.52	6.11	1.31
	% composition						24.3	59.0	12.7
	% kcal			30.5	**51.4**	15.5			

Supplementary Table 6. Macronutrient composition of the 3 different liquid mixed meals

HC: high carbohydrate, HF: high fat, and HP: high protein. MUFA: monounsaturated fatty acids, PUFA: polyunsaturated fatty acids, SFA: saturated fatty acids. As a benchmark for the high carbohydrate meal, we employed Ensure Plus® (1g = 1.41kcal, 0.05g protein, 0.045g fat, 0.1988g carbohydrate, 0.0057g SFA, 0.01095g MUFA, 0.02655g PUFA, 0g fiber), a product by Abbott Nutrition. For the high protein meal, we utilized Beneprotein® (1g powder = 3.57kcal, 0g fat, 0g carbohydrate, 0.85g protein, 5mg potassium, 5.7mg calcium, 2mg phosphorus, 0g fiber), manufactured by Nestlé Nutrition.
